# 

**Published:** 1917-06-23

**Authors:** 


### The Metropolitan Hospitals and Dispensaries.

**Table Showing the Sources of their Income in the Ten Years 1906 to 1915.**

| Year | From the Living | | From the Dead | | | | Total Income |
|---|---|---|---|---|---|---|---|
| | Subscriptions, Donations, Patients' payments, etc. | Percentage of Total | Investments | Percentage of Total | Legacies | Percentage of Total | |
| | £ | | | | £ | | £ |
| 1906 | 635,377 | 58 | 272,704 | 25 | 186,284 | 17 | 1,094.365 |
| 1907 | 653,320 | 48 | 296,218 | 22 | 407,918 | 30 | 1,357,456 |
| 1908 | 712.461* | 56 | 290,491 | 23 | 265,493 | 21 | 1,268,445 |
| 1909 | 701,458 | 57 | 294,206 | 24 | 240.523 | 19 | 1,236,187 |
| 1910 | 703,841 | 50 | 309.320 | 22 | 394,478 | 28 | 1,407,639 |
| 1911 | 738,622 | 53 | 329,253 | 24 | 322.949 | 23 | 1,390,824 |
| 1912 | 741,439 | 55 | 338,425 | 25 | 272,090 | 20 | 1,351,954 |
| 1913 | 845,908* | 52 | 349,295 | 22 | 424 339 | 26 | 1,619.542 |
| 1914 | 801.239 | 49 | 366,841 | 23 | 451,569 | 28 | 1,619,649 |
| 1915 | 978,294 | 62 | 368,536 | 23 | 242,049 | 15 | 1,588,879 |

* Includes £72,565 and £103,970, the amounts received by the London Hospital in 1908 and 1913 respectively as the result of its Quinquennial Appeals in those years.

Rates of Subscription: Twelve months, 6/6; Six months, 4/-; three months, 2/-, post free to any address in the United Kingdom. Abroad, Twelve months, 8/8; Six months, 5/-; Three months, 2/6, post free. **THE HOSPITAL "Index" to Volume LXI., October 1916 to March 1917, is now ready, price 2½d. per copy, post free.** Address The Manager, The Hospital, "The Hospital" Building, 28/29 Southampton Street, Strand, London, W.C. 2.

